# Relevance of a Hypersaline Sodium-Rich Naturally Sparkling Mineral Water to the Protection against Metabolic Syndrome Induction in Fructose-Fed Sprague-Dawley Rats: A Biochemical, Metabolic, and Redox Approach

**DOI:** 10.1155/2014/384583

**Published:** 2014-02-05

**Authors:** Cidália Dionísio Pereira, Milton Severo, João Ricardo Araújo, João Tiago Guimarães, Diogo Pestana, Alejandro Santos, Rita Ferreira, António Ascensão, José Magalhães, Isabel Azevedo, Rosário Monteiro, Maria João Martins

**Affiliations:** ^1^Department of Biochemistry (U38/FCT), Faculty of Medicine, University of Porto, 4200-319 Porto, Portugal; ^2^Department of Clinical Epidemiology, Predictive Medicine and Public Health, Faculty of Medicine, University of Porto, 4200-319 Porto, Portugal; ^3^Department of Clinical Pathology, São João Hospital Centre, EPE, 4200-319 Porto, Portugal; ^4^Faculty of Nutrition and Food Sciences, University of Porto, 4200-465 Porto, Portugal; ^5^QOPNA, Mass Spectrometry Centre, Department of Chemistry, University of Aveiro, 3810-193 Aveiro, Portugal; ^6^CIAFEL, Research Centre in Physical Activity, Health and Leisure, Faculty of Sport, University of Porto, 4200-450 Porto, Portugal

## Abstract

The Metabolic Syndrome increases the risk for atherosclerotic cardiovascular disease and type 2 Diabetes Mellitus. Increased fructose consumption and/or mineral deficiency have been associated with Metabolic Syndrome development. This study aimed to investigate the effects of 8 weeks consumption of a hypersaline sodium-rich naturally sparkling mineral water on 10% fructose-fed Sprague-Dawley rats (Metabolic Syndrome animal model). The ingestion of the mineral water (rich in sodium bicarbonate and with higher potassium, calcium, and magnesium content than the tap water used as control) reduced/prevented not only the fructose-induced increase of heart rate, plasma triacylglycerols, insulin and leptin levels, hepatic catalase activity, and organ weight to body weight ratios (for liver and both kidneys) but also the decrease of hepatic glutathione peroxidase activity and oxidized glutathione content. This mineral-rich water seems to have potential to prevent Metabolic Syndrome induction by fructose. We hypothesize that its regular intake in the context of modern diets, which have a general acidic character interfering with mineral homeostasis and are poor in micronutrients, namely potassium, calcium, and magnesium, could add surplus value and attenuate imbalances, thus contributing to metabolic and redox health and, consequently, decreasing the risk for atherosclerotic cardiovascular disease.

## 1. Introduction

The Metabolic Syndrome (MS) consists of multiple and interrelated risk factors of metabolic origin that appear to directly promote the development of atherosclerotic cardiovascular disease. The MS strongly associates with type 2 Diabetes Mellitus, or the risk for this condition. Although the exact etiology of the MS still remains unclear, it is known to involve complex interactions between genetic, metabolic, and environmental factors, where diet is of central importance [1–3].

There has been a substantial increase in fructose consumption, in the last decades, which has been associated with some adverse metabolic changes similar to those observed in the MS [4–7]. On the other hand, minerals like potassium, calcium, and magnesium, proposed as protective against the MS, are generally deficient in MS-inducing diets [3, 8–10].

Natural mineral waters are waters of underground origin, protected from contamination and microbiologically wholesome. They are characterized by their purity at source, content in minerals, trace elements, and other constituents as well as by favorable effects on human health [11]. Additionally, bioavailability of minerals from natural mineral waters is high [12–14].

The fructose-fed rat is an interesting and well-validated animal model of diet-induced MS (predominantly acquired MS model) that is commonly used in MS research [15]. Different rat strains with distinct fructose ingestion protocols are reported in the literature and, in all cases, fructose has been observed to induce MS features such as moderate hypertension, glucose intolerance, hyperinsulinemia, insulin resistance, dyslipidemia (hypertriglyceridemia, hypercholesterolemia), altered cytokine and adipokine status (altered tumor necrosis factor-alpha (TNF-*α*) and leptin levels, e.g.), decreased melatonin production, and/or increased body fat and/or body weight [15–20].

Both in humans and rats, a strong association has been found between MS and oxidative stress [21] and fructose-feeding associates with modification of the hepatic redox status [16, 22–24].

Beneficial effects of acute or chronic natural mineral-rich waters ingestion on blood pressure (BP) [25–27], metabolic profile (plasma insulin sensitivity [28], fasting serum glucose concentration [29], and fasting serum lipid profile [25, 29]), and plasma oxidative stress markers (reactive oxygen species [30], lipid and protein oxidation product levels, total antioxidant capacity, and total thiol levels [31]) have been published, but, to our knowledge, not in MS individuals or animal models. The natural mineral waters tested are rich, albeit in different proportions, in bicarbonate, calcium, magnesium, potassium, and/or sodium.

We aimed to investigate the possible beneficial effect of natural mineral-rich water on MS induction by fructose-feeding. In the present work, Sprague-Dawley rats (SDR) were fed with 10% fructose in natural mineral-rich water (Pedras Salgadas) for 8 weeks and compared to animals fed 10% fructose in tap water. The natural mineral-rich water tested has high total mineralization content (2855 mg/L), being mainly rich in sodium and bicarbonate and with higher potassium, calcium, and magnesium content than tap water.

## 2. Material and Methods 

### 2.1. Animals and Treatments

The study was carried out in 21 adult male CD SDR (388–483 g), from Charles River Laboratories (Chatillon/Chalaronne, France). Telemetry transmitters (TA11PA-C40, Data Sciences International (DSI), St. Paul, MN, USA) were implanted in the abdominal cavity, with the catheter in the abdominal aorta, by Charles River. Rats were shipped on the 5th day after surgery. Upon arrival, rats were individually housed, in an enriched environment, and maintained on a daily photoperiod of 12 h lighting schedule (20–22°C) with free access to standard laboratory pellet food (2014 Teklad Global 14% Protein Rodent Maintenance Diet from Harlan Interfauna Iberica SA, Barcelona, Spain) and tap water. Acclimatization took place for 10 days before starting the experimental protocol, which was authorized by the Veterinary National Department of the Ministry of Agriculture, Rural Development and Fisheries. During acclimatization, rats spent gradually increasing periods of time inside metabolic cages. The handling and care of the animals were conducted in conformity with the European Community Council guidelines for the use of experimental animals (86/609/EEC) and Act 129/92.

**Figure 1:**
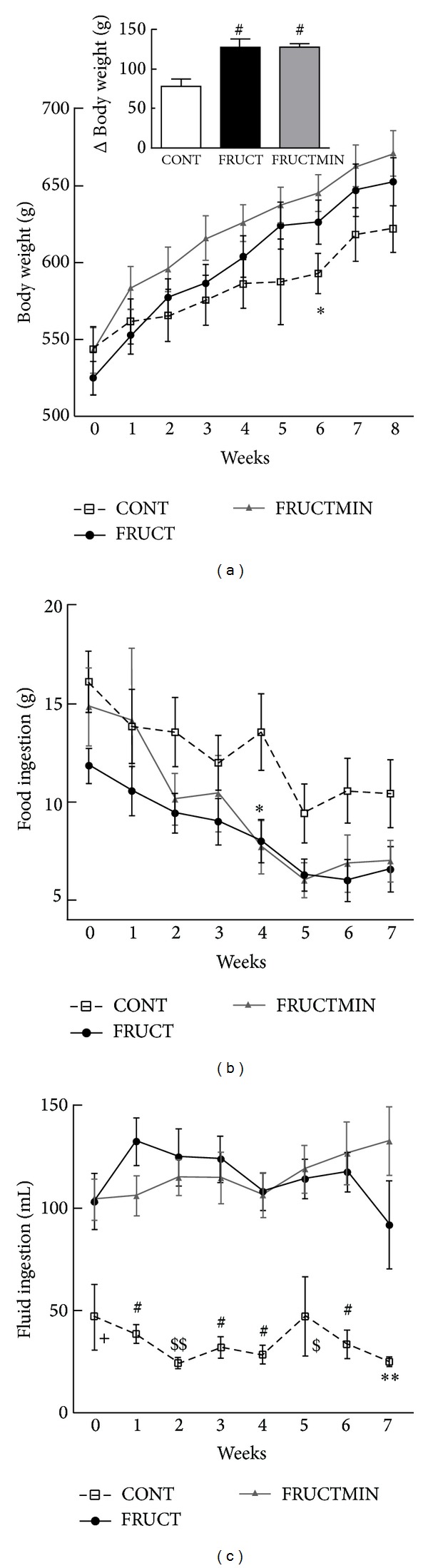
(a) Body weight (g; *n* = 7; *P < 0.05 CONT versus FRUCTMIN (Δ body weight (g; *n* = 7), between weeks 8 and 0 in the inset; ^#^ *P* < 0.01 FRUCT and FRUCTMIN versus CONT)), (b) food ingestion (g; *n* = 7; *P < 0.05 CONT versus FRUCTMIN, and (c) fluid ingestion evolution (mL; *n* = 7; ^+^ *P* < 0.05 CONT versus FRUCT and FRUCTMIN; ^#^ *P* < 0.001 CONT versus FRUCT and FRUCTMIN; ^$^ *P* < 0.05 CONT versus FRUCT and *P* < 0.01 CONT versus FRUCTMIN; ^$$^ *P* < 0.01 CONT versus FRUCT and *P* < 0.05 CONT versus FRUCTMIN; **P < 0.01 CONT versus FRUCTMIN), during the dietary intervention. Results were expressed as mean ± standard error of the mean. CONT: control; FRUCT: 10% fructose in tap water; FRUCTMIN: 10% fructose in natural mineral-rich water.

Animals were randomly divided into 3 groups (7 animals each) with free access to different drinking solutions: (a) tap water (CONT), (b) 10% fructose in tap water (FRUCT), or (c) 10% fructose in natural mineral-rich water (FRUCTMIN). All experimental groups were fed *ad libitum* with the standard laboratory chow diet mentioned above (20% of energy derived from protein, 13% from fat, and 67% from carbohydrate). The dietary manipulation lasted 8 weeks. A 3-week pretreatment period with the natural mineral-rich water was performed to the FRUCTMIN group (while the other rats were drinking tap water) to allow adjustment to water flavor and sparkles. This period of time induced no change in the pattern of food intake or increase of animals body weight (data not shown; all animals weighed 475–597 g at the beginning of the dietary manipulation with fructose). Body weight and food and fluid ingestion values were registered weekly, from week 0 to week 7 or 8 (as shown in Figures 1(a)–1(c), resp.). On week 0 these parameters were evaluated on the same day, which occurred 24 to 48 h after starting the dietary manipulation, and then on every one week after the first measurement. Each week, from week 0 to 7, all animals spent 24 h inside metabolic cages for evaluation of food and fluid consumption as well as for urine collection (the latter at 0, 2, 4, and 6 weeks). At each occasion, the three groups of rats were represented, with an equal number of animals per group. Total energy ingestion was calculated by multiplying food and fluid ingestion values by the corresponding reference energy values and then adding these two results.

The chemical characteristics of tap and natural mineral-rich waters are given in Table 1. The latter is classified as a hypersaline sodium-rich naturally sparkling mineral water, in conformity with the European Community Council guidelines for natural mineral waters (2009/54/EEC), and was kindly provided by Unicer Bebidas, SA (Leça do Balio, Matosinhos, Portugal).

### 2.2. Assessment of Blood Pressure and Heart Rate

Dataquest ART 4.1 Silver telemetry system (DSI) with RCP-1 receivers (and APR-1 for ambient pressure reference) was used for telemetric measurement of BP (mm Hg) and heart rate (HR; beats/min) in the animal cage. Dataquest ART acquisition software (DSI) was used to monitor all rats, for 8 weeks. Sampling was performed every min and setting segment duration at 20 s; 3 different animals (one from each experimental group) were evaluated per day from 4:00 p.m. to 8:00 a.m., seven days per week. BP values were not included/considered if pulse pressure (the difference between systolic and diastolic BP) was below 20 mm Hg and HR values were not used if pulse pressure values were lower than 10 mm Hg [32]. Data were exported from Dataquest ART 4.0 analysis program (DSI) to Microsoft Excel 2010 (Redmond, WA, USA) and then subjected to statistical analysis.

### 2.3. Collection of Samples

All chemical substances used in all the experiments were of analytical grade.

At the end of the dietary intervention, animals were deeply anesthetized with sodium pentobarbital (80 mg/kg of body weight) and blood was collected from the left ventricle into heparinized syringes. Then, rats were transcardially perfused with ice-cold isotonic sodium chloride solution. After perfusion, liver, heart, kidneys, and epididymal adipose tissue were rapidly removed from the thoracic and abdominal cavities, washed in cold saline solution, placed in qualitative filter paper for excess liquid removal, and weighed. The liver was cut into several fragments that were immersed in liquid nitrogen and stored at −80°C, until further processing.

### 2.4. Assessment of Plasma Biochemical, Metabolic, Hormonal, and Inflammatory Markers and Assessment of Urinary Creatinine and Sodium

Plasma concentrations of glucose, triacylglycerols, total cholesterol, HDL-cholesterol, LDL-cholesterol, C-reactive protein (CRP), glutamic-oxaloacetic transaminase (GOT), glutamic-pyruvic transaminase (GPT), total bilirubin, uric acid, urea, creatinine, total proteins, albumin, ferritin, sodium, potassium, chloride, magnesium, calcium, and phosphorus were determined. Urinary creatinine and sodium excretions were also evaluated. All these quantifications were made at the Clinical Pathology Unit of São João Hospital Centre, EPE, Porto, Portugal, using standardized methods for human sample routine hospital measurements.

Plasma levels of insulin (Mercodia AB, 10-1137-01 (Uppsala, Sweden)), adiponectin (Invitrogen Corporation, KRP0041 (Camarillo, CA, USA)), aldosterone (Uscn Life Science Inc., E0911Ra (Wuhan, China)), substance P (R&D Systems Inc., KGE007/SKGE007/PKGE007 (Minneapolis, MN, USA)), interleukin-6 (IL-6; Cusabio Biotech Co. Ltd., CSB-E04640r (Wuhan, China)), and TNF-*α* (Cusabio, CSB-E11987r) were evaluated according to the manufacturers' instructions from the specific ELISA kits. Plasma concentrations of melatonin (RSH69 K), nuclear factor kappa-B ligand (RANKL; RBN-31 K-1RANKL), leptin, and osteoprotegerin (OPG; RBN1-31 K) were measured with a Luminex 200 analyzer (Luminex Corporation, Austin, TX, USA) according to protocols (MILLIPLEX MAP kits) of Millipore Corporation (Billerica, MA, USA). Raw data (mean fluorescence intensity) were analyzed using ISTM 2.3 software (Luminex Corporation).

### 2.5. Assessment of Hepatic Redox State Markers

Oxidative damage to lipids, proteins, and DNA was evaluated by measuring thiobarbituric acid-reactive substances (viz., malondialdehyde (MDA)), carbonyls, and 8-hydroxy-2′-deoxyguanosine (8-OHdG) levels, respectively. Catalase, total superoxide dismutase (SOD), glutathione-S-transferase (GST), glutathione-peroxidase (GPx), and glutathione-reductase (GR) activities were quantified. Reduced (GSH) and oxidized (GSSG) glutathione concentrations were also determined. All these techniques were performed as described by Assunção et al. [33], except for the use of Bradford method for protein quantification and the use of a kit for 8-OHdG quantification (the DNA extraction kit (V-gene) was purchased from Bioron International (Ludwigshafen, Germany) and the 8-OHdG kit from Japan Institute for the Control of Aging (Haruoka, Fukuroi, Shizuoka, Japan)).

#### 2.5.1. Protein Extraction and Sirtuin 3 Protein Expression by Western Blot

Liver tissue samples (300–450 mg) were homogenized with a Teflon-glass homogenizer in an equal volume of protein extraction buffer (50 mM Tris-base, 150 mM NaCl, pH 7.4, 1% Triton X-100, 0.5% sodium deoxycholate, 0.1% sodium dodecyl sulfate (SDS), 1 mM EDTA, tablets of protease inhibitors, and phosphatase inhibitors (100 mM sodium fluoride and 10 mM sodium orthovanadate)), with subsequent agitation for 30 min at 4°C. Then, each sample was centrifuged, at 13 000 g for 20 min at 4°C, and the protein solution under the lipid layer was collected and kept at −80°C, until further analysis.

Proteins were quantified by using the bicinchoninic acid protein assay kit (Pierce, Rockford, IL, USA). Proteins were dissolved (1 : 1) in loading buffer (50 mM Tris-HCl, pH 6.8, 100 mM dithiothreitol, 2% SDS, 0.01% bromophenol blue and 10% glycerol) and denatured, for 5 min at 95°C. Then, 40 *μ*g of each sample was loaded per well, separated by electrophoresis in a 12% SDS polyacrylamide gel, and transferred to a nitrocellulose membrane (Hybond C-Extra, Amersham, GE Healthcare, Buckinghamshire, UK). The membrane was blocked in Tris-base-buffered saline with 0.1% Tween 20 (v/v) (TBST) containing 5% bovine serum albumin (w/v) and incubated overnight with the primary antibody against sirtuin 3 (Sirt3; Cell Signaling Technology Inc., Danvers, MA, USA) diluted 1 : 1500 in TBST, with gentle agitation, at 4°C. Then, the membrane was washed in TBST and incubated with donkey anti-rabbit polyclonal antibody conjugated to horseradish peroxidase (Santa Cruz Biotechnology Inc., Heidelberg, Germany), diluted 1 : 5000 in TBST, for 1 h at room temperature. Detection was performed with an enhanced chemiluminescence reagent (Amersham, GE Healthcare, Buckinghamshire, UK). Band intensity was determined using Image Lab software (version 4.0.1; Bio-Rad Laboratories, Hercules, CA, USA) and normalized for *β*-actin expression (1 : 1000 and 1 : 2000 for primary and secondary antibodies (Santa Cruz Biotechnology Inc., Heidelberg, Germany), respectively, diluted in 5% (w/v) of nonfat dry powdered milk Sveltesse (Nestlé Portugal SA, Linda-a-Velha, Portugal) in TBST).

### 2.6. Liver Magnesium and Calcium Content

Liver magnesium and calcium content were measured by inductively coupled plasma optical emission spectrometry (ICP-OES; ActivaM, JobinYvon, Horiba Scientific, Edison, NJ, USA), at 285.213 nm and 422.673 nm, respectively, according to ISO 11885 (water quality—determination of selected elements by ICP-OES (https://www.astandis.at/shopV5/Preview.action;jsessionid=CF7234FBAC2BCFDD35A4593A11BD4700?preview=&dokkey=347061&selectedLocale=en)), after microwave oven (Mars 5, CEM Corporation, Matthews, NC, USA) assisted acid digestion of liver fragments according to EPA 3052 (microwave assisted acid digestion of siliceous and organically based matrices (http://www.epa.gov/osw/hazard/testmethods/sw846/pdfs/3052.pdf)).

### 2.7. Statistical Methods

The significance of differences of each week, *cross-sectional statistical analysis*, among groups regarding systolic and diastolic BP, HR, body weight, food and fluid ingestions, urine volume, urinary sodium and creatinine excretions, total energy ingestion, and percentage energy supplied by fluid/total energy ingestion was evaluated using ANOVA followed by Bonferroni's multiple comparison test or by Kruskal-Wallis followed by Dunn's multiple comparison test, according to their distribution. These statistical methods were also used for evaluation of significance of differences among groups regarding organ weight/body weight, hepatic oxidative stress markers, and mineral content as well as plasma biochemical, metabolic, hormonal, and inflammatory data, at the end of the dietary intervention. The association between the outcomes (systolic and diastolic BP, HR, body weight, food and fluid ingestions, urine volume, urinary sodium and creatinine excretions, total energy ingestion, and percentage energy supplied by fluid/total energy ingestion) and the interaction of dietary intervention with time evolution (evaluated in weeks), *longitudinal statistical analysis*, was measured with the interaction terms (*β*), which were estimated by mixed effects model with random effect in the intercept. The area under the curve (AUC) was calculated through linear interpolation using the composite trapezoid rule [34].

Statistical analysis was performed using R: A language and environment for statistical computing [34], GraphPad Prism software (version 6.00; La Jolla, CA, USA), or IBM SPSS Statistics software (version 20.0; Armonk, NY, USA). Values were presented as mean ± standard error of the mean and differences considered significant for *P* < 0.05.

## 3. Results 

### 3.1. Parameters Evaluated over the 8-Week Period of Dietary Intervention

#### 3.1.1. Body Weight, Food and Fluid Ingestions

In general, on each separate week (week by week), body weight (Figure 1(a)) and food ingestion (Figure 1(b)) revealed similar values for the 3 animal groups. With time (over the dietary intervention period), a significantly higher and similar increase of body weight for both fructose groups versus CONT group was observed (see Supplementary Table 1 available online at http://dx.doi.org/10.1155/2014/384583 and inset in Figure 1(a)). With time FRUCTMIN rats decreased food ingestion significantly more than CONT rats and showed a trend towards a higher decrease with time than FRUCT rats (Supplementary Table 1). Week by week, fluid ingestion showed significantly higher values for both fructose-fed groups versus CONT group, without any significant difference between FRUCT and FRUCTMIN (Figure 1(c)), which was in accordance with AUC values (Supplementary Table 2). Fluid ingestion increased significantly for FRUCTMIN versus CONT and FRUCT with time (Supplementary Table 1).

#### 3.1.2. Energy Supplied by Fluid to Total Energy Ingestion Ratio and Total Energy Ingestion

**Figure 2:**
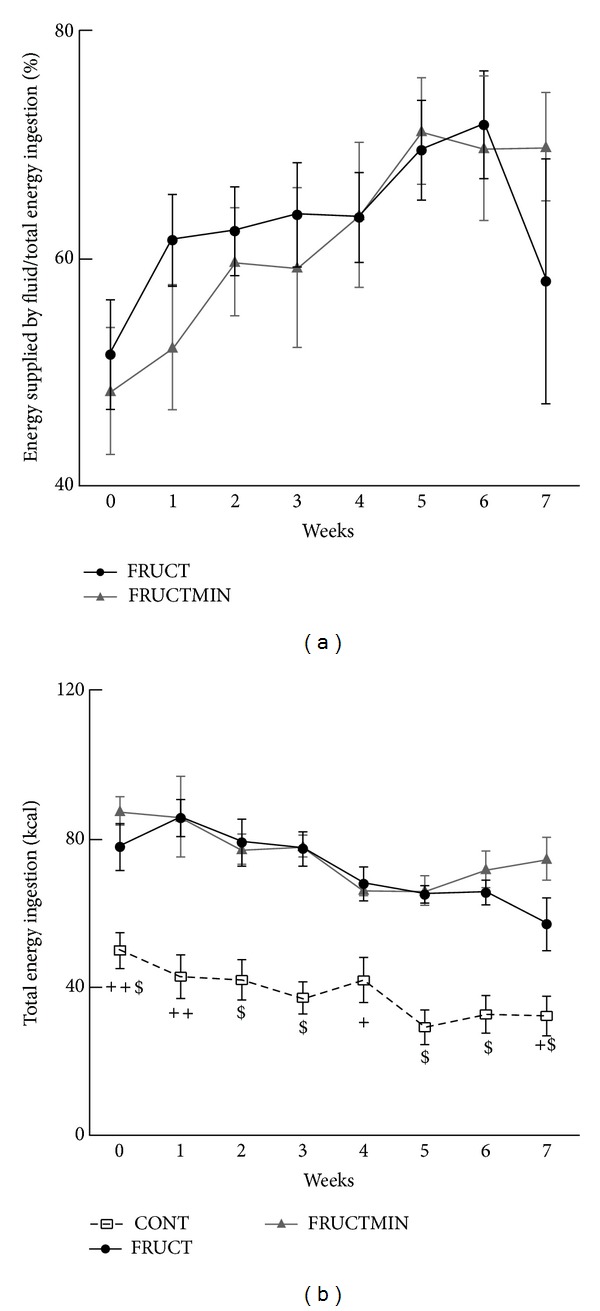
(a) Percentage energy supplied by fluid/total energy ingestion (*n* = 7) and (b) total energy ingestion evolution (kcal; *n* = 7; ^+^ *P* < 0.05 CONT versus FRUCT and FRUCTMIN; ^++^ *P* < 0.01 CONT versus FRUCT and FRUCTMIN; ^$^ *P* < 0.001 CONT versus FRUCT and FRUCTMIN; ^+$^ *P* < 0.05 CONT versus FRUCT and *P* < 0.001 CONT versus FRUCTMIN; ^++$^ *P* < 0.01 CONT versus FRUCT and *P* < 0.001 CONT versus FRUCTMIN), during the dietary intervention. Results were expressed as mean ± standard error of the mean. CONT: control; FRUCT: 10% fructose in tap water; FRUCTMIN: 10% fructose in natural mineral-rich water.

Week by week, no differences were observed for fructose ingestion (either from fluid ingestion or from both food and fluid ingestions (data not shown)) neither for percentage energy supplied by fluid/total energy ingestion between the two intervention groups (the latter being in accordance with AUC values; Figure 2(a) and Supplementary Table 2), in which there was a substantial proportion of energy ingested from fluid (48–72% of total energy ingestion; Figure 2(a)). Total energy ingestion was significantly higher every week of the protocol for both fructose-fed groups versus CONT group, without any significant difference between FRUCT and FRUCTMIN (Figure 2(b)), which was in accordance with AUC (Supplementary Table 2) and body weight (Figure 1(a)) results. Total energy ingestion decreased similarly with time for all groups (Supplementary Table 3; data not shown for controls).

#### 3.1.3. Urine Volume and Urinary Sodium and Creatinine Excretions

**Figure 3:**
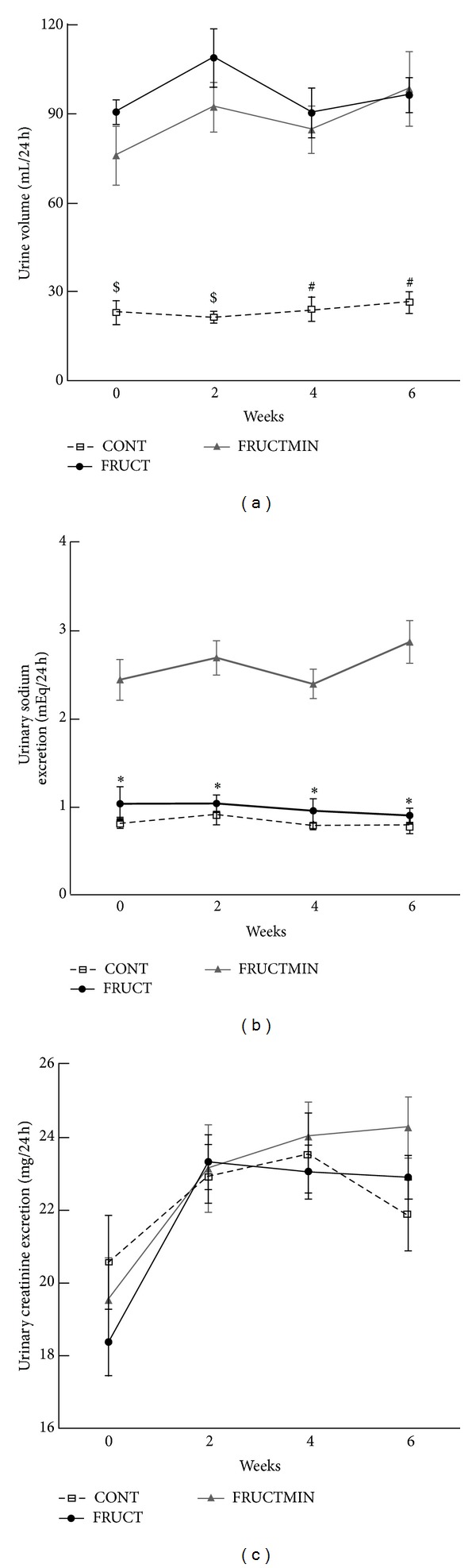
(a) Urine volume (mL/24 h; *n* = 7; ^$^ *P* < 0.01 CONT versus FRUCT and *P* < 0.05 CONT versus FRUCTMIN; ^#^ *P* < 0.01 CONT versus FRUCT and FRUCTMIN), (b) urinary sodium excretion (mEq/24 h; *n* = 6-7; *P < 0.01 FRUCTMIN versus CONT and *P* < 0.05 for FRUCTMIN versus FRUCT), and (c) urinary creatinine excretion evolution (mg/24 h; *n* = 6-7), every other week during the first 6 weeks of the dietary intervention. Results were expressed as mean ± standard error of the mean. CONT: control; FRUCT: 10% fructose in tap water; FRUCTMIN: 10% fructose in natural mineral-rich water.

With time, urine volume was significantly higher in FRUCTMIN versus CONT and FRUCT (Supplementary Table 4). Week by week, urinary sodium excretion values were expected when taking into consideration the sodium content of tap and natural mineral-rich waters: FRUCTMIN group had significantly higher values than the other two animal groups, without any significant difference between FRUCT and CONT (Figure 3(b)), which also agreed with AUC values (Supplementary Table 2).

#### 3.1.4. Blood Pressure and Heart Rate

**Figure 4:**
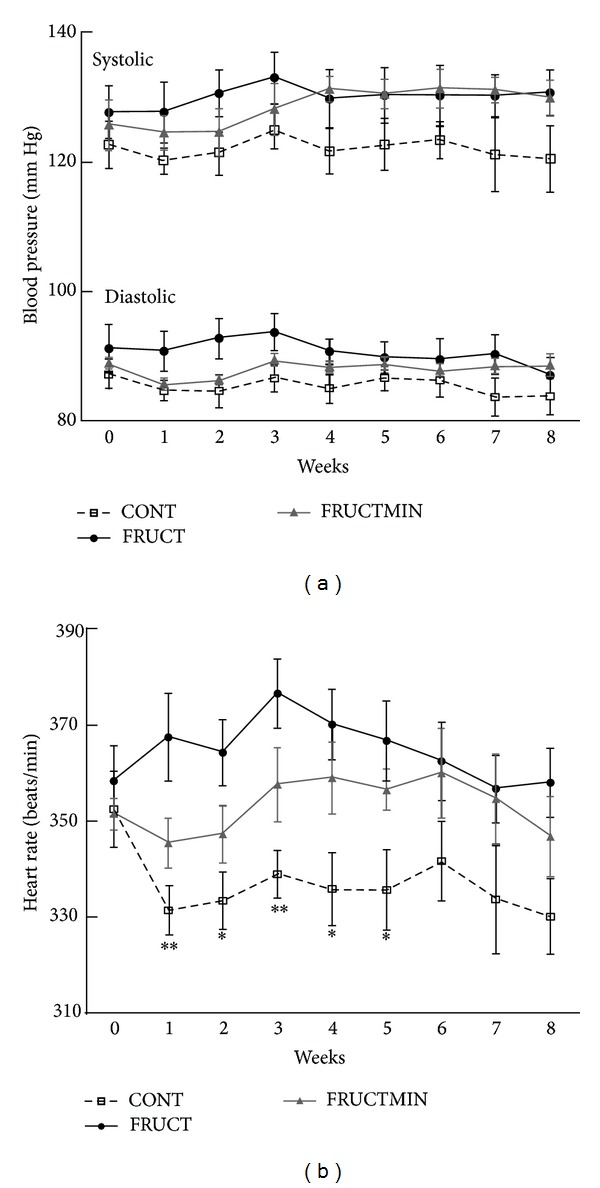
(a) Systolic blood pressure (mm Hg; *n* = 4–7) and diastolic blood pressure (mm Hg; *n* = 4–7), and (b) heart rate evolution (beats/min; *n* = 4–7; *P < 0.05 CONT versus FRUCT; **P < 0.01 CONT versus FRUCT), during the dietary intervention. Results were expressed as mean ± standard error of the mean. CONT: control; FRUCT: 10% fructose in tap water; FRUCTMIN: 10% fructose in natural mineral-rich water.

Between weeks 1 and 5, FRUCT rats had a significantly higher HR than CONT rats (Figure 4(b)). Interestingly, both systolic and diastolic BP and HR evolution over time seemed to be protected from fructose effects by the natural mineral-rich water until approximately half of the dietary intervention period (Figures 4(a)-4(b), resp.). A significant increase of systolic BP with time for both fructose groups versus CONT group was observed (Supplementary Table 5). Diastolic BP in FRUCTMIN group showed a tendency to increase with time versus CONT group (Supplementary Table 5). A significant increase of HR with time for FRUCTMIN versus CONT was observed (Supplementary Table 5).

### 3.2. Organ Weight to Body Weight Ratios

**Figure 5:**
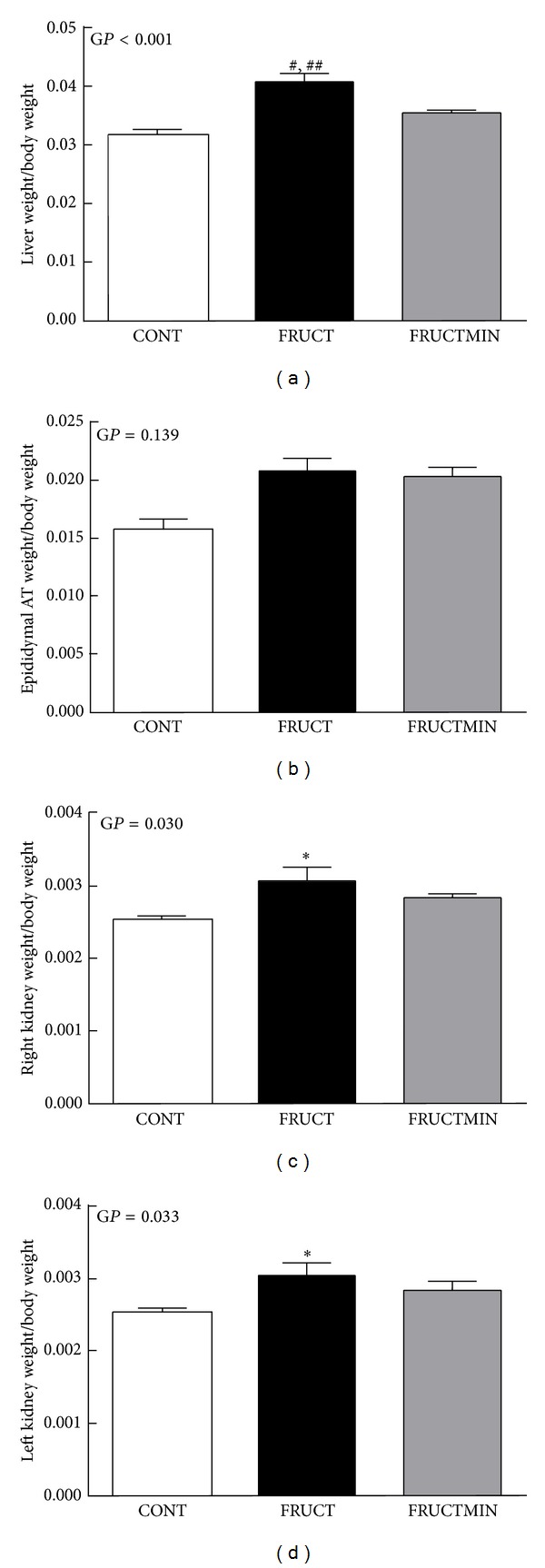
Organ weight to body weight ratios. (a) Liver (*n* = 7), (b) epididymal adipose tissue (*n* = 7), (c) right kidney (*n* = 6-7), and (d) left kidney (*n* = 6-7), at the end of the dietary intervention. Results were expressed as mean ± standard error of the mean. ^#^ *P* < 0.001 CONT versus FRUCT, ^##^ *P* < 0.005 FRUCT versus FRUCTMIN, and *P < 0.05 CONT versus FRUCT. AT: adipose tissue; CONT: control; FRUCT: 10% fructose in tap water; FRUCTMIN: 10% fructose in natural mineral-rich water; G*P*: global *P*.

Liver and both kidneys weight to body weight ratios were significantly higher in FRUCT versus CONT (Figures 5(a), 5(c), and 5(d), resp.). Additionally, the liver showed a strong trend to an increase in FRUCTMIN versus CONT (*P* = 0.053) and a significant increase in FRUCT versus FRUCTMIN (Figure 5(a)). Natural mineral-rich water ingestion prevented fructose effects on liver and both kidneys weight to body weight ratios.

Epididymal adipose tissue to body weight ratio was slightly and similarly higher in both fructose-fed animal groups versus CONT group (Figure 5(b)). No differences were found among groups regarding heart weight/body weight (data not shown).

### 3.3. Plasma Hormonal and Metabolic Profiles

**Figure 6:**
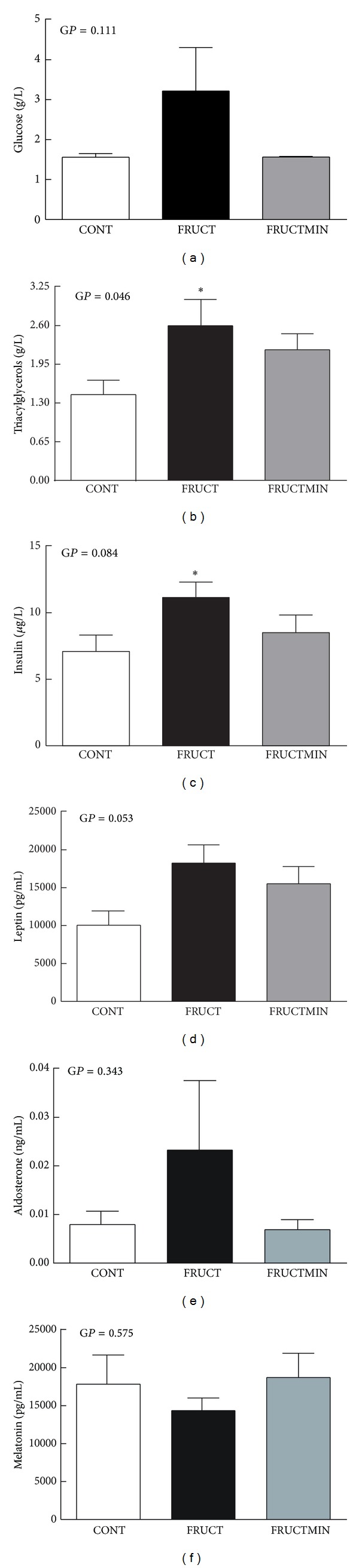
Metabolic markers and hormonal status in plasma. (a) Glucose (g/L; *n* = 7), (b) triacylglycerols (g/L; *n* = 5–7), (c) insulin (*μ*g/L; *n* = 7), (d) leptin (pg/mL; *n* = 6), (e) aldosterone (ng/mL; *n* = 7), and (f) melatonin levels (pg/mL; *n* = 6), at the end of the dietary intervention. Results were expressed as mean ± standard error of the mean. *P < 0.05 CONT versus FRUCT. CONT: control; FRUCT: 10% fructose in tap water; FRUCTMIN: 10% fructose in natural mineral-rich water; G*P*: global *P*.

Triacylglycerol levels significantly increased in FRUCT versus CONT and a tendency to an increase in FRUCTMIN versus CONT (*P* = 0.080) was observed (Figure 6(b)). Insulin significantly increased (Figure 6(c)) and leptin variation followed the same pattern in FRUCT versus CONT (*P* = 0.057) (Figure 6(d)). Insulin sensitivity index was also calculated [35] and a strong tendency to a decrease was observed in FRUCT versus CONT (*P* and global *P* = 0.055; 0.247 × 10^6^ ± 0.032 × 10^6^, 0.137 × 10^6^ ± 0.009 × 10^6^, and 0.211 × 10^6^ ± 0.030 × 10^6^ for CONT, FRUCT, and FRUCTMIN, resp.). Glucose (Figure 6(a)) and aldosterone (Figure 6(e)) seemed to increase and melatonin (Figure 6(f)) seemed to decrease in FRUCT versus CONT. Natural mineral-rich water ingestion appeared to counteract these fructose-induced metabolic and hormonal effects. No variations were observed for adiponectin levels (data not shown).

### 3.4. Plasma Biochemical and Inflammatory Profiles

Urea (Table 2) and magnesium (Table 3) levels significantly decreased in the two fructose-fed groups versus the CONT group. Total proteins and albumin levels significantly increased in both groups of fructose-fed animals versus CONT group (except for total proteins in FRUCTMIN versus CONT where a strong tendency was observed) (Table 2). TNF-*α* and IL-6 levels seemed to increase and OPG to RANKL ratio seemed to decrease in FRUCT versus CONT (Table 2), with the natural mineral-rich water improving these parameters. CRP and substance *P* levels slightly increased in FRUCTMIN versus the other two animal groups (Table 2). The replacement of food by fructose solution as an energy source could explain the similar decreases in plasma urea, magnesium, GOT, GPT, ferritin, and uric acid levels in both fructose-fed SDR groups versus CONT group (although significantly only for some parameters).

### 3.5. Hepatic Redox Status Markers

**Figure 7:**
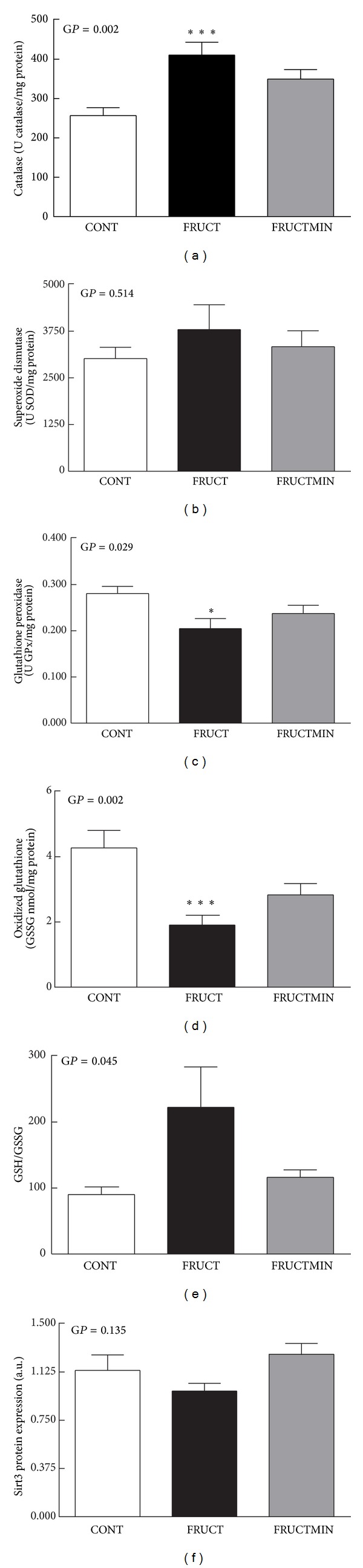
Redox state markers in liver. (a) Catalase (U catalase/mg protein; *n* = 7), (b) superoxide dismutase (U SOD/mg protein; *n* = 7), (c) glutathione peroxidase activities (U GPx/mg protein; *n* = 7), (d) oxidized glutathione content (nmol/mg protein; *n* = 7), (e) GSH to GSSG ratio (*n* = 7), and (f) sirtuin 3 protein expression (arbitrary units (a.u.); *n* = 5-6), at the end of the dietary intervention. Results were expressed as mean ± standard error of the mean. *P < 0.05 CONT versus FRUCT; ***P < 0.005 CONT versus FRUCT. CONT: control; FRUCT: 10% fructose in tap water; FRUCTMIN: 10% fructose in natural mineral-rich water; G*P*: global *P*; GPx: glutathione peroxidase; GSH: reduced glutathione; GSSG: oxidized glutathione; Sirt3: sirtuin 3; SOD: superoxide dismutase.

Catalase and SOD activities and GSH to GSSG ratio increased (Figures 7(a), 7(b), and 7(e), resp.) and GPx activity, GSSG level and Sirt3 protein expression decreased (Figures 7(c), 7(d), and 7(f), resp.) in FRUCT versus CONT (significantly for catalase, GPx, and GSSG and a strong tendency for GSH/GSSG (*P* = 0.062, global *P* = 0.045)). Natural mineral-rich water ingestion counteracted all these modifications. Regarding catalase and GSSG, there was a strong trend to, respectively, a decrease and an increase in FRUCTMIN versus FRUCT (*P* = 0.065 and *P* = 0.055, resp.). No significant modifications were observed for 8-OHdG levels (data not shown) neither for other redox parameters (Table 4).

### 3.6. Liver Magnesium and Calcium Content

**Figure 8:**
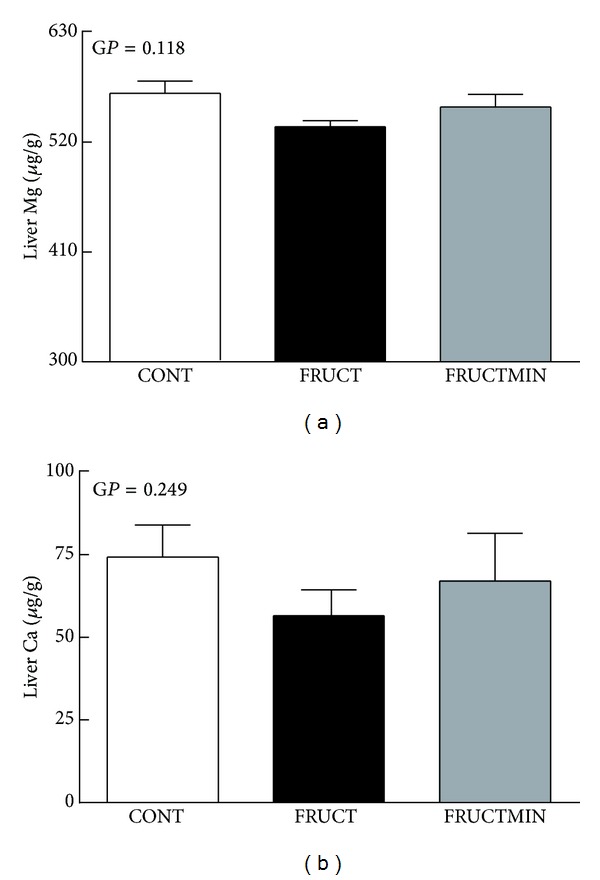
Liver magnesium (a) and calcium (b) content (*μ*g/g of tissue lyophilize; *n* = 6 for both elements), at the end of the dietary intervention. Results were expressed as mean ± standard error of the mean. Ca: calcium; CONT: control; FRUCT: 10% fructose in tap water; FRUCTMIN: 10% fructose in natural mineral-rich water; G*P*: global *P*; Mg: magnesium.

A slight decrease was observed in FRUCT versus CONT for both liver magnesium and calcium content that was prevented by natural mineral-rich water ingestion (Figures 8(a) and 8(b), resp.), most particularly for magnesium.

## 4. Discussion

The fructose-fed SDR model mimics a predominantly environmentally acquired MS model [15] that is commonly used in MS research. Similarly, in the present study, many of the alterations observed in different protocols of fructose-induced MS were recapitulated. Increased systolic BP, adiposity index and liver and kidney weight to body weight ratios as well as modulation of the hepatic redox status and similar changes in the plasma levels of hormones, except for aldosterone, and/or energy substrates evaluated in this work have been reported in different protocols of fructose-induced MS in SDR [15–18, 20, 22–24, 36–38].

As previously reported [17], fructose intervention increases SDR body weight, but besides fructose metabolic effects, two details of our experimental protocol could have contributed to body weight increase: rats were housed individually, which may have reduced their physical activity, and were already adult rats at the beginning of the dietary manipulation (their age was reflected in the high body weight values 475–597 g), which may have amplified fructose metabolic effects [39]. Additionally to the variations observed in food and fluid ingestions seen in the CONT group with aging (over the 8 weeks of dietary intervention), fructose-fed rats adjusted fluid and food ingestions, aiming to maintain the level of energy consumption, as previously described [18]. Fructose-fed animals increased their body weight similarly between them and more than control rats (associated with a small increase of epididymal body-fat), reflecting the absence of any major natural mineral-rich water consumption effect on both food and fructose ingestions. Accordingly, effects shown below against MS induction in FRUCTMIN rats related exclusively to natural mineral-rich water ingestion and, interestingly, natural mineral-rich water ingestion reduced/prevented the majority of the fructose effects and, consequently, protected against MS induction, which, to our knowledge, is described here for the first time. MS represents a risk for cardiovascular disease (whose prevalence is increasing worldwide), which, together with the recent report of Luo et al. on the consumption of low-mineral bottled water that increases the risk for cardiovascular disease [40], demonstrates the high relevance of our research.

The significant increase in plasma insulin levels in the FRUCT versus CONT group could have contributed to the significant effects in systolic BP and HR described before (the effect of natural mineral-rich water with time on HR and diastolic BP decreased after body weight adjustment). Hyperinsulinemia may increase BP and HR by increasing the sympathetic nervous system activity, through alteration in the neuronal vascular control and/or by enhancement of kidney sodium reabsorption [6, 41, 42]. Increased sympathetic modulation of vessels and heart precedes metabolic dysfunction in mice drinking 10% fructose in tap water for up to 2 months [43]. HR, a marker of autonomic dysfunction, associates with MS, particularly with insulin resistance, and interestingly, in Japan, the prevalence of MS increases linearly with the increase in HR [1, 44].

BP correlates with plasma aldosterone levels and an association between plasma aldosterone levels and hyperinsulinemia has been described in obesity [45]. Although both fructose-fed groups in the present study had significantly increased body weight versus controls, the insulin value in the FRUCTMIN group (that after body weight adjustment presented a strong tendency to decrease versus FRUCT (data not shown)), along with the later BP increase, was in accordance with the unaltered aldosterone levels in the FRUCTMIN group. In fructose-fed SDR, the absence of a significant increase in body weight associates with no increase of aldosterone levels, in spite of hyperinsulinemia [46].

Leptin resistance may be an early feature of metabolic dysfunction induced by fructose-feeding, since it may precede increased adiposity, elevated circulating leptin levels, and changes in glucose metabolism in rats. Despite higher leptin levels (with the comparison versus CONT significant after adjustment for body weight (data not shown)), which would anticipate a reduction in food intake and body fat in healthy conditions, FRUCT rats had a lower decrease of food ingestion with time than FRUCTMIN rats (significant after adjustment for body weight (data not shown)) as well as a similar weight gain and amount of epididymal fat. These results could reflect a phenomenon of selective leptin resistance that, together with the activation of the sympathetic nerves by hyperleptinemia, could have contributed to the earlier development of hypertension in FRUCT rats [5, 47–49]. Melatonin has anti-inflammatory, antihyperlipidemic, and antihypertensive properties and it is known to influence insulin secretion and to enhance its action (it increases insulin sensitivity and enhances insulin effects on leptin expression) [19, 50–52]. The apparent deregulation of leptin, melatonin, insulin, and aldosterone observed in the FRUCT group, owing to modifications in the hormone levels, was less evident in the FRUCTMIN group.

Fructose is highly lipogenic as its hepatic metabolism provides great amounts of triose phosphate precursors for fatty acid synthesis [5, 6]. Reduced leptin and insulin sensitivities contribute to hypertriglyceridemia induced by fructose ingestion [53]. The difference in triacylglycerol levels in the two fructose-fed groups could be explained by the improvement of leptin, insulin, and aldosterone levels in FRUCTMIN induced by the natural mineral-rich water ingestion (the magnitude of triacylglycerols increase versus CONT was reduced after body weight adjustment (data not shown)). Normalization of melatonin levels could have also contributed [52, 54].

The increase in plasma albumin and total protein levels has been described in fructose-fed rats [55], which could reflect a combination of undernutrition (also because of the decrease in food ingestion), some degree of liver disorder (resulting from MS induction), and/or dehydration (owing to loose stools resulting from incomplete fructose absorption) [56, 57]. Nevertheless, the pattern of urine volume mirrored the pattern of fluid ingestion and we did not observe loose stools, which makes dehydration unlikely in the fructose-fed rats.

Although we found a significant increase in both kidneys weight to body weight ratios in the FRUCT group, we believe that there was no renal functional alteration in this SDR group taking into consideration the plasma and/or urinary profiles of creatinine, urea, albumin, total proteins, magnesium, sodium, potassium, calcium, chloride, and phosphorous. Despite an increase in the kidney weight to body weight ratio, Rizkalla et al. reported no glomerular basement membrane thickening in SDR after 10 weeks of 57% fructose-feeding [36].

Fructose-feeding accelerates osteoporosis [58] and, accordingly, the OPG to RANKL ratio (reflecting the ratio of osteoblast versus osteoclast activities [58, 59]) seemed to decrease in the FRUCT group. Interestingly, and in accordance with FRUCT group results, TNF-*α* and IL-6 are important mediators in the process of osteoclast differentiation and activation [59] and, thus, the changes observed in their levels might have contributed to the lower OPG to RANKL ratio. High levels of leptin and aldosterone have been linked to proinflammatory and prooxidant actions [45, 47]. In the FRUCTMIN group, the improvement of the leptin, aldosterone, TNF-*α*, and IL-6 values could have contributed to the improvement of the OPG to RANKL ratio.

Taking into consideration all the results obtained for both fructose-fed SDR groups, the slightly increased levels of substance P and CRP in FRUCTMIN rats were unexpected. Plasma substance P levels increase under magnesium deficiency and contribute to increase inflammation and protein and lipid oxidation [60]. In fact, plasma magnesium levels of both fructose groups were significantly decreased, but the FRUCTMIN group displayed better plasma TNF-*α* and IL-6 levels as well as lower hepatic protein oxidation content (after adjustment for body weight this latter parameter showed a tendency to a decrease in FRUCTMIN versus the other two groups, most particularly versus FRUCT (data not shown)). Interestingly, high CRP levels occur in energy restricted animals [61].

In FRUCT animals, the absence of oxidative lesions in lipids, proteins, and DNA (the same as for proteins happened for DNA oxidative lesions (data not shown)) could be explained by the significantly increased catalase activity and apparently increased SOD activity. Polizio et al. describe similar results for lipid oxidative lesions and catalase and SOD activities by 10% fructose ingestion in tap water [24]. The significant decrease in GPx activity in FRUCT rats could be partially compensated by the significant increase in catalase activity, since both enzymes can eliminate hydrogen peroxide (converting it to water). Catalase is responsible for the elimination of high concentrations of hydrogen peroxide, while GPx does it when concentrations of hydrogen peroxide are low [62]. The higher levels of hydrogen peroxide could have resulted from the fructose-feeding [7, 63] and the small decrease of Sirt3 protein expression could have intensified reactive oxygen species production in FRUCT rats [64]. The significant decrease in hepatic GPx activity could have contributed to the strong tendency for an increase in the GSH/GSSG ratio in FRUCT versus CONT, by oxidizing less GSH to GSSG. Increased GSSG efflux from hepatocytes and/or increased hepatic GSH synthesis induced by fructose could also apply. However, we did not observe an increase in GSH level as it might have been expected from a lower GSH oxidation and/or increased GSH synthesis. Although cellular ATP depletion induced by fructose prevents ATP-dependent GSSG efflux in freshly isolated rat hepatocytes [65], this phenomenon should not have a strong impact here since an increase in uric acid formation was not observed. The variations observed for GPx, SOD, and catalase activities in the two fructose-fed groups are in accordance with the antioxidant actions of melatonin (probably its primary function) and Sirt3. Melatonin possesses free radical-scavenging activity, stimulates antioxidant enzymes (e.g., GPx), and inhibits reactive oxygen/nitrogen species producing enzymes [66, 67]. Sirt3 has beneficial effects on mitochondrial electron transport chain (contributing to a reduction in the production of reactive oxygen species) and mitochondrial antioxidant enzymes (probably also on GPx, like melatonin) [64]. Reduction of Sirt3 associates with an accelerated development of metabolic abnormalities similar to the MS [68], which is in agreement with our overall results.

Our results also showed that the natural mineral-rich water could contribute to the preservation of the hepatic intracellular ions, namely the magnesium content. It is well documented that plasma ion levels might not reflect their tissue levels, and here this was evident regarding plasma and hepatic concentrations of magnesium and calcium. Magnesium deficiency is associated with insulin resistance. For quite some time, it was thought that it could be the cause of insulin resistance, but very recently it was described that type 2 Diabetes Mellitus and a lower degree of metabolic control are essential in accounting for the lower levels of serum magnesium that occur in obese individuals [69].

## 5. Conclusion

**Figure 9:**
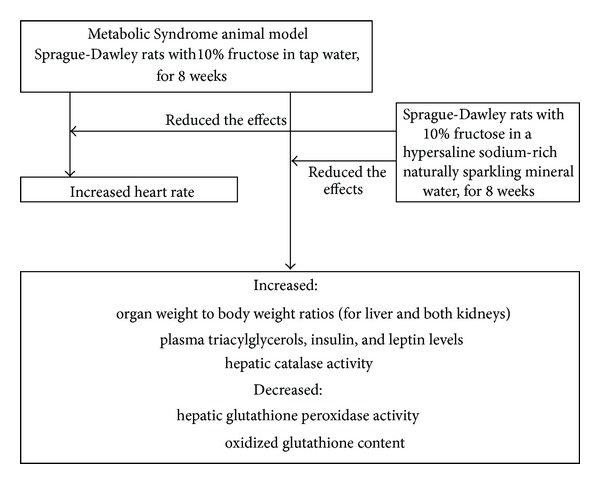
Summary of fructose-feeding effects that were reduced/prevented by the natural mineral-rich water.

Figure 9 summarizes the significant effects of fructose-feeding obtained in this research that were reduced/prevented by the natural mineral-rich water (taking into consideration that when fructose was coingested with the natural mineral-rich water no significant effects were observed versus the control).

Still, although for some of the parameters evaluated in our study the extension of differences among groups did not achieve statistical significance, the variations observed were consistent with the pattern expected, which reinforces their biological relevance and justifies their presentation and discussion.

The results here described suggest that this natural mineral-rich water seems to have potential to prevent MS induction. We hypothesize that its regular intake in the context of modern diets, which have a general acidic character interfering with mineral homeostasis and are poor in micronutrients, namely potassium, calcium, and magnesium, could add surplus value and attenuate imbalances, thus contributing to metabolic and redox health and, consequently, decreasing the risk for atherosclerotic cardiovascular disease.

## Supplementary Material

Longitudinal statistical analysis and area under curve results for all parameters evaluated over the 8 week period of dietary intervention with 10% fructose, either in tap water or in natural mineral-rich water.

## Figures and Tables

**Table 1:** Chemical characteristics of tap and natural mineral waters.

Characteristics	Tap water	Hypersaline sodium-rich naturally sparkling mineral water—Pedras Salgadas
Total mineralization (mg/L)	148–151	2855
pH	6.5–9.0	6.16
Sodium (mg/L)	200	591
Calcium (mg/L)	30.5–40.2	92.5
Magnesium (mg/L)	3.6–9.2	26.2
Potassium (mg/L)	2.6	29.9
Copper (mg/L)	2	0.0013
Zinc (µg/L)	∗	1.1
Selenium (µg/L)	10	<2.0
Bicarbonate (mg/L)	∗	2013
Chloride (mg/L)	250	30.8
Sulphate (mg/L)	250	6.4

∗: no need for control (Portuguese Act 306/2007, from 27th of August).

**Table 2:** Plasma biochemical and inflammatory marker levels, at the end of the dietary intervention.

	CONT		FRUCT		FRUCTMIN		Global *P* [*P* between two groups]
	Mean	(SEM)	Mean	(SEM)	Mean	(SEM)	
GOT (U/L), *n* = 7	105.14	(13.062)	82.14	(11.066)	84.71	(7.383)	0.279
GPT (U/L), *n* = 7	50.86	(8.681)	38.57	(1.192)	36.57	(2.626)	0.146
Total bilirubin (mg/L), *n* = 5–7	2.00	(0.0655)	1.90	(0.0447)	2.07	(0.127)	0.481
Uric acid (mg/L), *n* = 5–7	6.14	(0.662)	4.72	(0.371)	5.01	(0.403)	0.161
Urea (g/L), *n* = 5–7	0.286	(0.0104)	0.148	(0.0198)	0.186	(0.0210)	<0.001 [(C versus F) < 0.001; (C versus FM) 0.001; (F versus FM) 0.163]
Creatinine (mg/L), *n* = 5–7	5.14	(0.153)	4.56	(0.108)	5.00	(0.162)	0.052 [(C versus F) 0.019; (C versus FM) 0.492; (F versus FM) 0.065]
Total proteins (g/L), *n* = 5–7	59.57	(0.634)	62.98	(0.881)	61.49	(0.710)	0.018 [(C versus F) 0.006; (C versus FM) 0.068; (F versus FM) 0.182]
Albumin (g/L), *n* = 5–7	27.23	(0.342)	29.50	(0.214)	29.01	(0.304)	<0.001 [(C versus F) < 0.001; (C versus FM) 0.001; (F versus FM) 0.283]
Total cholesterol (g/L), *n* = 5–7	0.686	(0.0334)	0.666	(0.100)	0.753	(0.0390)	0.525
HDL-cholesterol (g/L), *n* = 5–7	0.357	(0.0224)	0.3540	(0.0486)	0.397	(0.0218)	0.518
LDL-cholesterol (g/L), *n* = 5–7	0.201	(0.0126)	0.204	(0.0227)	0.229	(0.0201)	0.505
HDL-cholesterol/total cholesterol, *n* = 5–7	0.520	(0.0140)	0.536	(0.0104)	0.527	(0.00888)	0.637
HDL-cholesterol/LDL cholesterol, *n* = 5–7	1.82	(0.189)	1.75	(0.164)	1.81	(0.171)	0.963
Ferritin (U/L), *n* = 5–7	24.33	(2.577)	19.18	(0.450)	19.63	(0.374)	0.082 [(C versus F) 0.055; (C versus FM) 0.055; (F versus FM) 0.859]
OPG (pg/mL), *n* = 6-7	789.57	(226.538)	571.21	(40.265)	667.87	(83.676)	0.606
RANKL (pg/mL), *n* = 6-7	10.15	(1.694)	13.91	(2.675)	7.79	(1.470)	0.143
OPG/RANKL, *n* = 5–7	126.37	(57.324)	45.37	(9.376)	109.38	(41.657)	0.401
C-reactive protein (mg/L), *n* = 5-6	0.04167	(0.0095)	0.03800	(0.0058)	0.05500	(0.0034)	0.216
Substance P (pg/mL), *n* = 7	1978.67	(184.475)	1968.55	(216.113)	2416.07	(158.299)	0.186
TNF-*α* (pg/mL), *n* = 7	718.80	(121.457)	2282.75	(1142.161)	818.77	(241.407)	0.216
IL-6 (pg/mL), *n* = 5-6	3.007	(0.412)	3.486	(0.601)	2.681	(0.363)	0.514

C or CONT: control; F or FRUCT: 10% fructose in tap water; FM or FRUCTMIN: 10% fructose in natural mineral-rich water; GOT: glutamic-oxaloacetic transaminase; GPT: glutamic-pyruvic transaminase; IL-6: interleukin-6; OPG: osteoprotegerin; RANKL: receptor activator of nuclear factor kappa-B ligand; SEM: standard error of the mean; TNF-*α*: tumor necrosis factor-alpha.

**Table 3:** Plasma electrolyte content, at the end of the dietary intervention.

	CONT		FRUCT		FRUCTMIN		Global *P* [*P* between groups]
	Mean	(SEM)	Mean	(SEM)	Mean	(SEM)	
Sodium (mEq/L), *n* = 5–7	142.42	(1.270)	143.60	(0.872)	144.57	(0.719)	0.318
Potassium (mEq/L), *n* = 5–7	5.90	(0.236)	5.92	(0.208)	5.48	(0.150)	0.261
Chloride (mEq/L), *n* = 5–7	100.00	(0.976)	99.60	(0.600)	100.29	(0.565)	0.836
Magnesium (mEq/L), *n* = 5–7	1.71	(0.0513)	1.54	(0.0385)	1.55	(0.0431)	0.025 [(C versus F) 0.020; (C versus FM) 0.017; (F versus FM) 0.873]
Calcium (mEq/L), *n* = 5–7	5.32	(0.0346)	5.44	(0.0600)	5.42	(0.0359)	0.124
Phosphorus (mg/L), *n* = 5–7	76.11	(3.841)	76.62	(1.964)	79.30	(3.167)	0.756

C or CONT: control; F or FRUCT: 10% fructose in tap water; FM or FRUCTMIN: 10% fructose in natural mineral-rich water; SEM: standard error of the mean.

**Table 4:** Hepatic redox state characterization, at the end of the dietary intervention.

	CONT		FRUCT		FRUCTMIN		Global *P *
	Mean	(SEM)	Mean	(SEM)	Mean	(SEM)	
Glutathione-reductase (U GR/mg protein), *n* = 7	0.0297	(0.0015)	0.0320	(0.0011)	0.0322	(0.0022)	0.507
Glutathione-S-transferase (U GST/mg protein), *n* = 7	0.3754	(0.0261)	0.3738	(0.0319)	0.3764	(0.0263)	0.998
Reduced glutathione (nmol/mg protein), *n* = 7	360.5082	(16.4242)	332.0008	(9.0570)	309.9596	(29.5796)	0.235
Malondialdehyde (nmol/mg protein), *n* = 6	0.0108	(0.0010)	0.0100	(0.0012)	0.0121	(0.0013)	0.424
Protein carbonyls (nmol/mg protein), *n* = 7	0.8465	(0.0732)	0.9828	(0.0808)	0.7305	(0.0866)	0.113

CONT: control; FRUCT: 10% fructose in tap water; FRUCTMIN: 10% fructose in natural mineral-rich water; GR: glutathione-reductase; GST: glutathione-S-transferase; SEM: standard error of the mean.
