# Supplementary material for: Relevance of a Hypersaline Sodium-Rich Naturally Sparkling Mineral Water to the Protection against Metabolic Syndrome Induction in Fructose-Fed Sprague-Dawley Rats: A Biochemical, Metabolic, and Redox Approach
Source: Int J Endocrinol. 2014 Feb 5;2014:384583. doi: 10.1155/2014/384583 (PMC3941211; doi:10.1155/2014/384583)
Supplement: Supplementary file 1 — Longitudinal statistical analysis and area under curve results for all parameters evaluated over the 8 week period of dietary intervention with 10% fructose, either in tap water or in natural mineral-rich water. [file 384583.f1.pdf]

Cidália Dionísio Pereira, Milton Severo, João Ricardo Araújo, João Tiago Guimarães, Diogo Pestana, Alejandro Santos, Rita Ferreira, António Ascensão, José Magalhães, Isabel Azevedo, Rosário Monteiro, Maria João Martins. Relevance of a hypersaline sodium-rich naturally sparkling mineral water to the protection against Metabolic Syndrome induction in fructose-fed Sprague-Dawley rats: a biochemical, metabolic and redox approach.

Longitudinal statistical analysis and area under curve results for all parameters evaluated over the dietary intervention with 10% fructose, either in tap water or in natural mineral-rich water.

Supplementary Table 1. Interaction terms (β), estimated by mixed-effects model with random effect in the intercept, for *longitudinal statistical analysis* of body weight and food and fluid ingestions.

Interaction terms (β) estimated by mixed-effects model with random effect in the intercept								
**Body weight (g)**	**β**	***P***	**Food ingestion (g)**	**β**	***P***	**Fluid ingestion (mL)**	**β**	***P***
**CONT**	**reference**		**CONT**	**reference**		**CONT**	**reference**	
**FRUCT**	**7.4218**	**< 0.001**	**FRUCT**	**-0.0357**	**0.8699**	**FRUCT**	**-1.0663**	**0.6015**
**FRUCTMIN**	**6.2925**	**< 0.001**	**FRUCTMIN**	**-0.4677**	**0.0334**	**FRUCTMIN**	**5.0085**	**0.0151**
**FRUCT**	**reference**		**FRUCT**	**reference**		**FRUCT**	**reference**	
**FRUCTMIN**	**-1.1293**	**0.3229**	**FRUCTMIN**	**-0.4320**	**0.0766**	**FRUCTMIN**	**6.0748**	**0.0034**

CONT: control; FRUCT: 10% fructose in tap water; FRUCTMIN: 10% fructose in natural mineral-rich water.

Supplementary Table 2. Area under the curve (AUC) values for all parameters evaluated over the dietary intervention.

	AUC			Ratios of AUC		
	**CONT**	**FRUCT**	**FRUCTMIN**	**FRUCT/ CONT**	**FRUCTMIN /CONT**	**FRUCTMIN /FRUCT**
**Body weight (g)**	4671	4808	4975	1.029	1.065	1.035
**Food ingestion (g)**	86.29	58.51	66.21	0.6781	0.7673	1.132
**Fluid ingestion (mL)**	240.2	819.4	806.9	3.411	3.359	0.9847
**% Energy supplied by fluid/total energy ingestion**		448.0	435.5			0.9721
**Total energy ingestion (kcal)**	267.5	509.1	528.0	1.903	1.974	1.037
**Urinary volume (mL/24h)**	140.6	586.0	528.9	4.168	3.762	0.9026
**Urinary sodium excretion (mEq/24h)**	5.033	5.941	15.54	1.180	3.088	2.616
**Urinary creatinine excretion (mg/24h)**	135.5	134.1	138.2	0.9897	1.020	1.031
**Systolic blood pressure (mm Hg)**	977.5	1042	1030	1.066	1.054	0.9885
**Diastolic blood pressure (mm Hg)**	683.2	726.6	704	1.064	1.030	0.9689
**Heart rate (beats/min)**	2692	2923	2830	1.086	1.051	0.9682

CONT: control; FRUCT: 10% fructose in tap water; FRUCTMIN: 10% fructose in natural mineral-rich water. Results presented without standard error of the mean, as in M. Bialer, T. Shekh-Ahmad, T. L. Braun, and M. B. Halvorsen, “Comparative steady-state pharmacokinetic evaluation of immediate-release topiramate and USL255, a once-daily extended-release topiramate formulation,” Epilepsia vol. 54, no. 8, pp. 1444-1452, 2013.

Supplementary Table 3. Interaction terms (β), estimated by mixed-effects model with random effect in the intercept, for *longitudinal statistical analysis* of percentage energy supplied by fluid/total energy ingestion and total energy ingestion.

**Interaction terms (β) estimated by mixed-effects model with random effect in the intercept**					
**% Energy supplied by fluid/total energy ingestion**	**β**	***P***	**Total energy ingestion (kcal)**	**β**	***P***
			**CONT**	**reference**	
			**FRUCT**	**-1.0726**	**0.2344**
			**FRUCTMIN**	**0.0182**	**0.9839**
**FRUCT**	**reference**		**FRUCT**	**reference**	
**FRUCTMIN**	**1.5899**	**0.1473**	**FRUCTMIN**	**1.0908**	**0.2979**

CONT: control; FRUCT: 10% fructose in tap water; FRUCTMIN: 10% fructose in natural mineral-rich water.

Supplementary Table 4. Interaction terms (β), estimated by mixed-effects model with random effect in the intercept, for *longitudinal statistical analysis* of urine volume and urinary sodium and creatinine excretions.

Interaction terms (β) estimated by mixed-effects model with random effect in the intercept								
**Urine volume (mL/24h)**	**β**	***P***	**Urinary sodium excretion (mEq/24h)**	**β**	***P***	**Urinary creatinine excretion (mg/24h)**	**β**	***P***
**CONT**	**reference**		**CONT**	**reference**		**CONT**	**reference**	
**FRUCT**	**-2.0059**	**0.1242**	**FRUCT**	**-0.0112**	**0.7673**	**FRUCT**	**0.4324**	**0.1255**
**FRUCTMIN**	**3.5520**	**0.0070**	**FRUCTMIN**	**0.0534**	**0.1633**	**FRUCTMIN**	**0.5046**	**0.0748**
**FRUCT**	**reference**		**FRUCT**	**reference**		**FRUCT**	**reference**	
**FRUCTMIN**	**5.5578**	**< 0.001**	**FRUCTMIN**	**0.0650**	**0.1605**	**FRUCTMIN**	**0.0665**	**0.7941**

CONT: control; FRUCT: 10% fructose in tap water; FRUCTMIN: 10% fructose in natural mineral-rich water.

Supplementary Table 5. Interaction terms (β), estimated by mixed-effects model with random effect in the intercept, for *longitudinal statistical analysis* of systolic and diastolic blood pressure and heart rate.

Interaction terms (β) estimated by mixed-effects model with random effect in the intercept								
**Systolic BP (mm Hg)**	**β**	***P***	**Diastolic BP (mm Hg)**	**β**	***P***	**Heart rate (beats/min)**	**β**	***P***
**CONT**	**reference**		**CONT**	**reference**		**CONT**	**reference**	
**FRUCT**	**0.7598**	**0.0042**	**FRUCT**	**0.2438**	**0.1948**	**FRUCT**	**1.2247**	**0.1274**
**FRUCTMIN**	**0.4875**	**0.0469**	**FRUCTMIN**	**0.3347**	**0.0572**	**FRUCTMIN**	**1.5640**	**0.0377**
**FRUCT**	**reference**		**FRUCT**	**reference**		**FRUCT**	**reference**	
**FRUCTMIN**	**-0.2758**	**0.2611**	**FRUCTMIN**	**0.0949**	**0.6324**	**FRUCTMIN**	**0.3547**	**0.6706**

CONT: control; FRUCT: 10% fructose in tap water; FRUCTMIN: 10% fructose in natural mineral-rich water.
